# Enhancing geomechanical characteristics of calcium sulfoaluminate (CSA) cement-treated soil under low confining pressures

**DOI:** 10.1038/s41598-024-61548-8

**Published:** 2024-05-21

**Authors:** James Innocent Ocheme, Jong Kim, Sung-Woo Moon

**Affiliations:** Department of Civil and Environmental Engineering, Nazarbayev University, 53 Kabanbay Batyr Ave, 010000 Astana, Kazakhstan

**Keywords:** Calcium sulfoaluminate cement, Soil improvement, Low confining pressure, Quartz sand, Shear strength, Triaxial test, Civil engineering, Mechanical properties

## Abstract

This study examines the efficacy of employing calcium sulfoaluminate (CSA) cement, an environmentally friendly binder, for enhancing the geomechanical characteristics of sand, particularly under low confining pressure conditions. A series of triaxial consolidated drained tests were performed on sand samples treated with varying content (5, 7, and 10%) of CSA cement and 10% ordinary Portland cement (OPC) under various low confining pressures (50, 100, 200, and 400 kPa). The test findings demonstrated the importance of cement content and confining pressure on the mode of failure, stress–strain and volumetric behavior, failure characteristics, and shear strength parameters of the treated quartz sand. After a curing period of 14 days, samples treated with 10% CSA cement exhibited a remarkable 212% increase in peak deviator stress and an 89% reduction in axial strain at failure, indicating higher initial stiffness compared to untreated samples under a 400 kPa confining pressure. Furthermore, the samples treated with 10% CSA exhibited higher peak deviator stress, initial stiffness, and strength development compared to those treated with 10% OPC. The scanning electron microscopy analysis provides insights into particle breakage and bond degradation processes, which increase with confining pressure in CSA-treated samples. Also, the mode of failure analysis reveals a transition from ductile to slightly brittle behavior with increasing cement content. Notably, the geomechanical properties of the treated material emphasized the significant impact of CSA cement on soil improvement. Thus offering a sustainable alternative for soil improvement in construction projects.

## Introduction

Historically, cementitious materials, including fly ash, lime, and ordinary Portland cement (OPC), have been employed to ameliorate the geomechanical properties of soils. This soil treatment approach enhances foundation base stability, durability, and load-bearing capacity, thus offering superior foundations for construction projects^1^. Moreover, fortifying soil strength by applying cementitious materials becomes imperative with the increasing global population and the pressing need for infrastructure development. For instance, in sandy regions grappling with challenges such as low load-bearing capacity and high permeability, soil improvement becomes essential for augmenting foundation stability and load-bearing capacity while mitigating settlement concerns.

Furthermore, OPC has become the most widely used cementitious material among the traditional cementitious materials used for soil improvement. Although it offers considerable benefits, it also possesses notable disadvantages, including an exceedingly high carbon footprint. According to the study conducted by Andrew^2^, the degradation of carbonates in making Portland cement accounts for about 8% of global anthropogenic carbon dioxide (CO_2_) emissions. However, in search of an alternative binder, many researchers have recently utilized industrial waste substances, such as rice husk ash, blast furnace slag, and fly ash, to enhance the engineering properties of soils^3–7^. Similarly, several researchers studied the importance of using sustainable cementitious materials like calcium sulfoaluminate (CSA) cement for soil improvement instead of OPC^8–11,32–35^.

The main benefit of CSA cement over OPC is its lesser CO_2_ emissions due to the presence of ye'elimite^12^. According to the study by Nie, et al.^13^, CSA cement has a reduced carbon footprint, emitting 34% less CO_2_ during the production process than OPC cement. Thus, CSA is a viable eco-friendly alternative for OPC as it aids in minimizing greenhouse gas emissions^14,15^. Furthermore, many research investigations have indicated that CSA cement has a significant potential for soil improvement, notably in terms of quick setting time, its ability to withstand freeze–thaw cycles, and its resistance to sulfate attacks^9,10,15,16,36,37^.

Pooni et al.^8^ reported the benefit of CSA cement on the mechanical and microstructural properties of soil. The research findings indicated that CSA cement can effectively enhance soil's mechanical properties while reducing carbon emissions associated with using traditional cementitious materials for soil improvement.

Owing to its rapid setting time and fast increase in strength development, CSA cement would be suitable for work with short deadlines and unfavorable weather conditions. Thus, CSA cement provides an environmentally friendly solution to the environmental problems associated with Portland cement.

Hence, in addressing geotechnical challenges, it is imperative to recognize that numerous engineering issues manifest under low confining pressure. Consequently, the geomechanical behavior of soil significantly varies under conditions of low confining pressure compared to those at moderate or high pressures. Existing research on soil improvement with cement has primarily focused on soil behavior under moderate to low confining pressures^17–23^. However, it is noteworthy that most of this research pertains to soil improvement using OPC as the stabilizing agent, leaving a notable gap in understanding CSA-treated sand behavior under low confining pressure. This gap is particularly concerning as low confining pressure scenarios are common in practical settings, such as foundations with shallow depths and embankments on soils. Moreover, no study has investigated the collective impact of low confining pressure and CSA cement on the mechanical behavior and shear strength of cemented sand. Hence, the objectives of this study are to (1) examine the triaxial behavior of sand treated with CSA cement under low confining pressure; (2) analyze the geomechanical properties exhibited by sand treated with both OPC and CSA cement; (3) investigate the influence of confining pressure on the deformation characteristics, failure mechanism, and mechanical properties of cemented sand, with consolidated drained triaxial tests.

## Materials and experimental work

**Figure 1:**
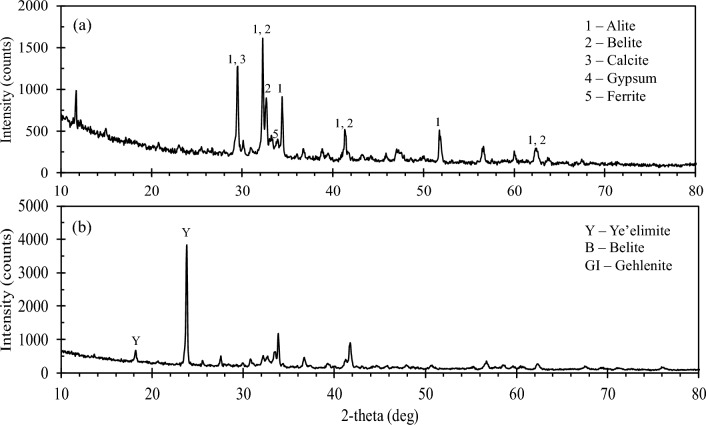
XRD investigation results (**a**) OPC, (**b**) CSA.

The quartz sand employed in this research exhibits a coefficient of uniformity and curvature of 1.46 and 0.96, respectively. According to the USCS classification based on ASTM/D2487-17e1^24^, the sand was categorized as SP, indicating that it is poorly graded. The physical properties of the sand is depicted in Table 1. Additionally, type 1 OPC and CSA cement served as cement binders in this investigation. X-ray diffraction (XRD) findings of the two cement types are presented in Fig. 1. The XRD results reveal that the primary components of CSA cement include gehlinite, belite, and ye'elimite. Similarly, OPC cement primarily consists of alite, belite, calcite, gypsum, and ferrite. Subramanian, et al.^16^ reported that substituting 30% of CSA cement with gypsum led to a substantial increase in the early strength of sand, with a continuous improvement in strength when used for soil improvement. Consequently, a portion of the CSA content in this study was replaced with gypsum at the optimal amount of 30%.

**Table 1:** Physical properties of the quartz sand.

Properties	Value
Effective diameter (D_10_) (mm)	0.65
Effective diameter (D_60_) (mm)	0.95
Coefficient of curvature Cc	1.46
Coefficient of uniformity Cu	0.96
Specific gravity	2.64
USCS	SP

The sand used for sample preparation was mixed with 5%, 7%, and 10% CSA cement. García et al.^38^ observed a significant increase in the UCS of sand when 3 to 11% OPC was added. Thus, a separate mixture with 10% OPC was prepared to evaluate and compare the soil behavior with CSA cement. The sand and cement were blended for approximately five minutes using a Hobart mixer until a homogeneous consistency was achieved. Subsequently, clean water was added to the mix and stirred for an additional ten minutes, following the procedure outlined in ASTM/D698^25^. The optimum moisture contents (OMCs) of sand-cement mixtures are similar to those obtained by Ocheme et al.^26^. The mixed material was compacted in three layers within a 76 mm by 38 mm cylindrical mold. Before compaction, oil was applied to the inner surfaces of the molds to facilitate specimen extraction. Each of the three layers was compacted 25 times using a rammer. To avoid complications associated with smooth compaction planes and ensure adequate surface-to-surface contact, the upper portion of the first compacted layer was sacrificed before placing and compacting the subsequent layer, following the recommendation by Ding et al.^27^.

Following compaction, the specimens were wrapped in a thin membrane layer and stored at room temperature for curing. After three days, the specimens were extruded, and testing was conducted to assess early strength development. The remaining samples were sealed in a plastic membrane to prevent moisture loss and stored for the remainder of the curing time. Following the ASTM/D7181-20^28^ standard, consolidated drained triaxial tests were conducted on the remaining specimens after 7 and 14 days of curing.

**Figure 2:**
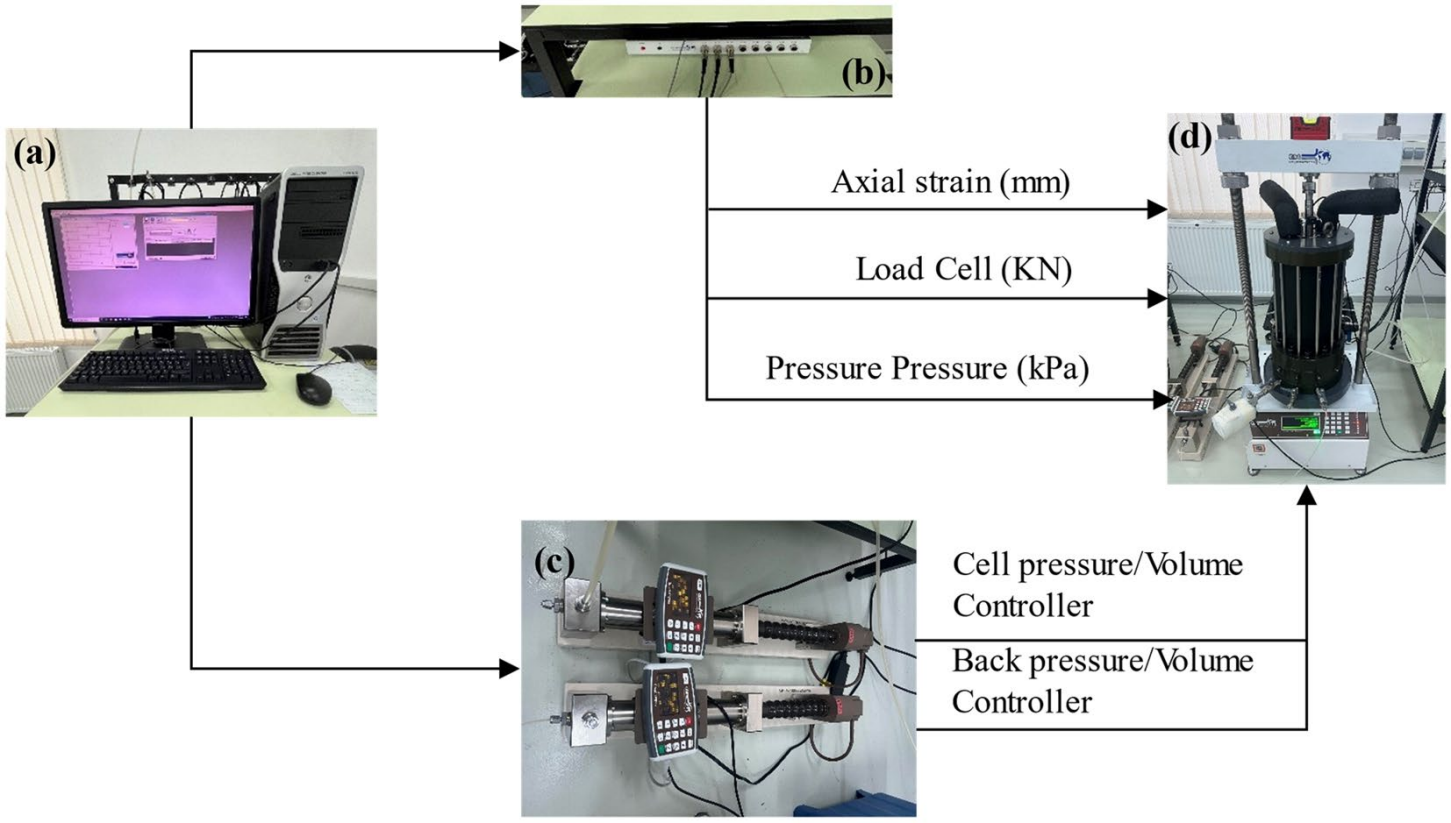
ETAS components (**a**) GDSLAB software, (**b**) data logger, (**c**) cell and back pressure controller, (**d**) an image of the GDS triaxial system in the loading frame.

This study employed the environmental triaxial automated system (ETAS), developed by GDS Instruments, for triaxial testing*.* Figure 2 illustrates the essential components of the triaxial system. The ETAS utilized in this research is equipped with a triaxial cell and pressure controller capable of managing pressures up to 4 MPa and loads up to 50 kN, respectively. Preceding testing, the ETAS underwent flushing using the back pressure/volume controller to remove any entrapped air. De-aired water served as the pore fluid, while silicon oil was employed as the cell oil. The test material was positioned on a base pedestal, with filter papers and two porous stones positioned above and below it. After placing the samples on the base pedestal, a layer of membrane with an average thickness of 0.3 mm was applied to protect them from the compressed chamber oil. Additionally, to prevent cell oil from infiltrating the sample, two ring seals were positioned around the base pedestal and on top of the sample. The complete triaxial cell assembly was then filled with oil. To achieve saturation, the test samples underwent a three-hour flushing with de-aired water, maintaining a back pressure of 10 kPa lower than the confining pressure. Subsequently, both the cell and back pressures were incrementally increased until Skempton's B-value reached 0.95 or higher. The sample was subjected to a B-check to confirm the saturation level after the saturation period. The B-check parameter can be defined by the equation below:

1 $$B = \frac{{\Delta {\text{u}}}}{{\Delta \sigma_{C}^{{\prime }} }}$$

where ∆u is the change in PWP due to a rise in confining pressure, and ∆σ’_c_ is the change in confining pressure.

The test specimens were consolidated to cell pressures (σ’_c_) of 50 kPa, 100 kPa, 200 kPa, and 400 kPa and subsequently subjected to shearing under draining conditions. Shearing was accomplished by applying a constant axial strain at a rate of 0.1 mm per minute. Axial loading continued until a 20% axial strain was achieved. Deviator stresses were recorded at 60-s intervals throughout the shearing process.

## Results and discussion

### Stress–strain and volumetric strain behavior

Table 2 presents the results of the tests conducted on CSA-treated samples at the failure and ultimate state conditions. The deviator stress, q (kPa), and mean effective stress, p′ (kPa), are defined by the equation below:

2 $${{\text{q}}}= {\mathrm{\sigma}}^{\prime}_{1}- {\mathrm{\sigma}}^{\prime}_{3}$$

3 $${{\text{p}}}^{{{\prime}}}= \frac{{(\mathrm{\sigma}}^{\prime}_{1}+ {2\mathrm{\sigma}}^{\prime}_{3})}{3}$$

where σ′_1_ denotes the effective axial stress (kPa), and σ′_3_ denotes the effective radial stress (kPa).

**Table 2:** Summary of the CD triaxial tests results for CSA-treated sand.

Test identification	Initial state			Failure condition				Ultimate condition			
	Confining pressure, kPa	Curing period, days	Cement content, %	Peak deviator stress at failure, kPa	Mean effective stress at failure, kPa	Peak friction angle, º	Peak cohesion, kPa	Ultimate deviator stress, kPa	Ultimate mean effective stress, kPa	Ultimate friction angle, º	Ultimate cohesion, kPa
CD-0/0.05	50	–	0	203	112	32	44	131	89	24	31
CD-0/0.1	100	–	0	323	203	32	44	198	162	24	31
CD-0/0.2	200	–	0	608	398	32	44	440	343	24	31
CD-0/0.4	400	–	0	1048	744	32	44	603	596	24	31
CD-5/0.05/7	50	7	5	586	256	32	141	191	123	25	45
CD-5/0.1/7	100	7	5	824	384	32	141	349	226	25	45
CD-5/0.2/7	200	7	5	911	514	32	141	432	353	25	45
CD-5/0.4/7	400	7	5	1419	884	32	141	735	654	25	45
CD-5/0.05/14	50	14	5	802	313	29	219	349	162	27	90
CD-5/0.1/14	100	14	5	867	385	29	219	340	209	27	90
CD-5/0.2/14	200	14	5	1104	563	29	219	571	386	27	90
CD-5/0.4/14	400	14	5	1484	890	29	219	909	386	27	90
CD-7/0.05/7	50	7	7	838	340	34	203	241	141	30	44
CD-7/0.1/7	100	7	7	1235	522	34	203	292	207	30	44
CD-7/0.2/7	200	7	7	1512	714	34	203	483	371	30	44
CD-7/0.4/7	400	7	7	1768	999	34	203	977	734	30	44
CD-7/0.05/14	50	14	7	1333	491	36	343	206	114	33	54
CD-7/0.1/14	100	14	7	1388	558	36	343	339	208	33	54
CD-7/0.2/14	200	14	7	2027	871	36	343	627	402	33	54
CD-7/0.4/14	400	14	7	2396	1195	36	343	1086	757	33	54
CD-10/0.05/3	50	3	10	1727	622	49	339	147	94	36	28
CD-10/0.1/3	100	3	10	3032	1107	49	339	435	240	36	28
CD-10/0.2/3	200	3	10	3329	1305	49	339	878	488	36	28
CD-10/0.4/3	400	3	10	4484	1889	49	339	1092	759	36	28
CD-10/0.05/7	50	7	10	2525	887	49	444	291	142	40	52
CD-10/0.1/7	100	7	10	3106	1131	49	444	377	221	40	52
CD-10/0.2/7	200	7	10	3429	1338	49	444	706	430	40	52
CD-10/0.4/7	400	7	10	4735	1973	49	444	1546	911	40	52
CD-10/0.05/14	50	14	10	2837	991	46	570	242	127	42	46
CD-10/0.1/14	100	14	10	2955	1081	46	570	397	228	42	46
CD-10/0.2/14	200	14	10	3444	1345	46	570	645	411	42	46
CD-10/0.4/14	400	14	10	4792	1993	46	570	1751	980	42	46

The "CD" stands for the "consolidated drained" triaxial testing in the CD-A/B/C. "A" represents the amount of cement, "B" denotes the confining pressure, and "C" signifies the curing time.

**Figure 3:**
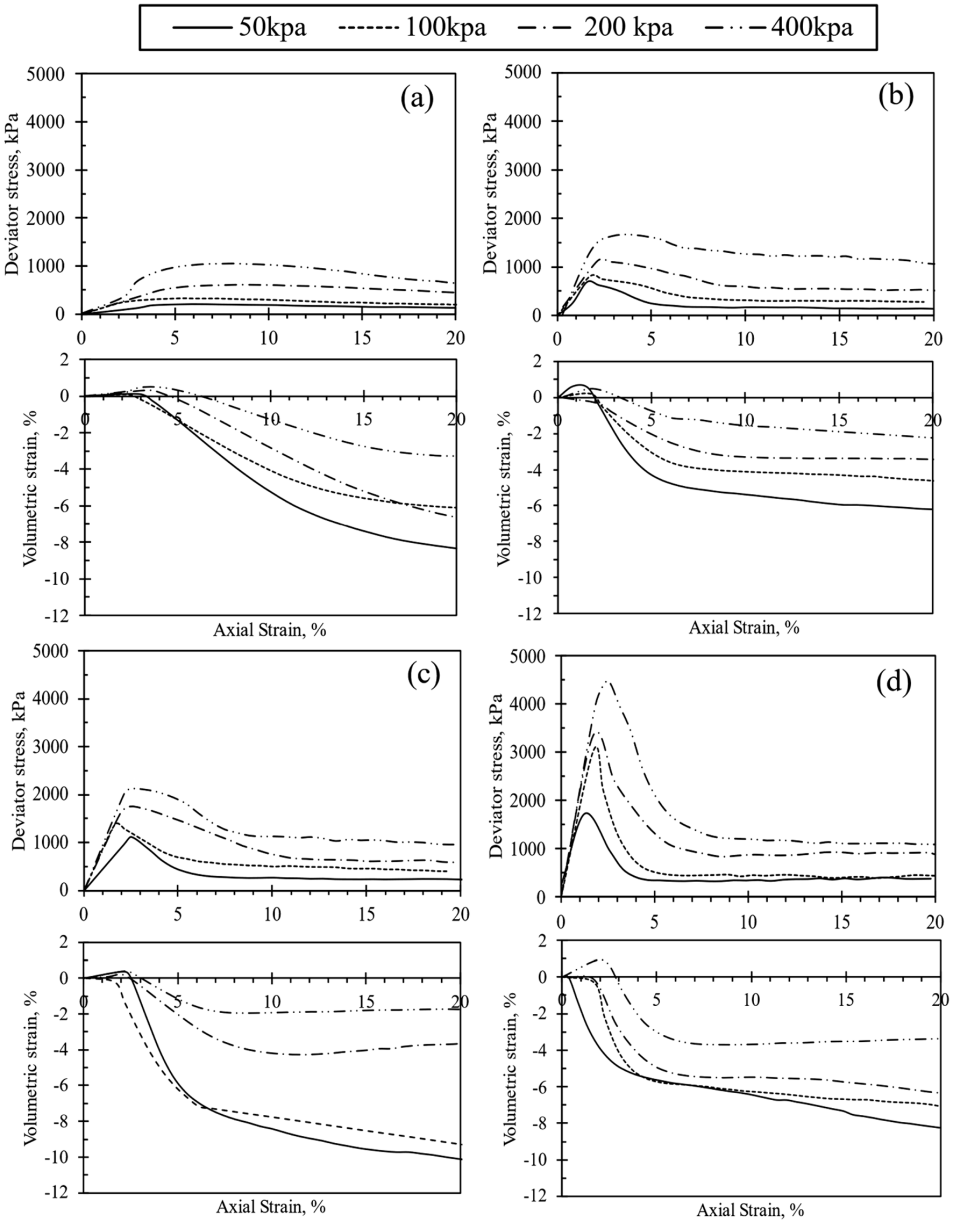
Stress–strain and volumetric-strain curves of CSA cement-treated samples after 3 days of curing, represented for varying cement content: (**a**) untreated soil, (**b**) 5%, (**c**) 7%, and (**d**) 10%.

**Figure 4:**
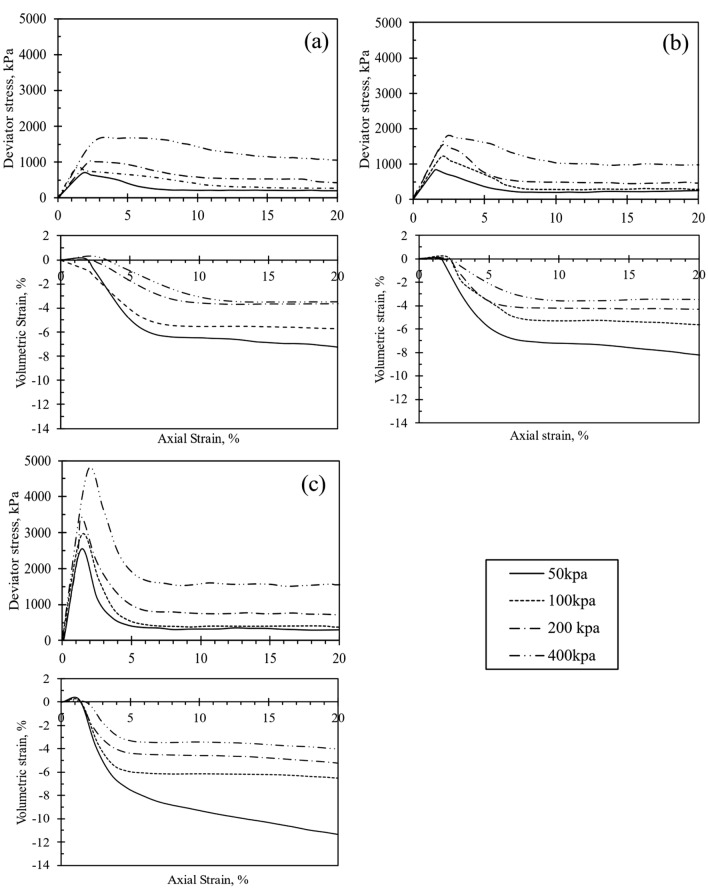
Stress–strain and volumetric-strain curves of CSA cement-treated samples after 7 days of curing, represented for varying cement content: (**a**) 5%, (**b**) 7%, and (**c**) 10%.

**Figure 5:**
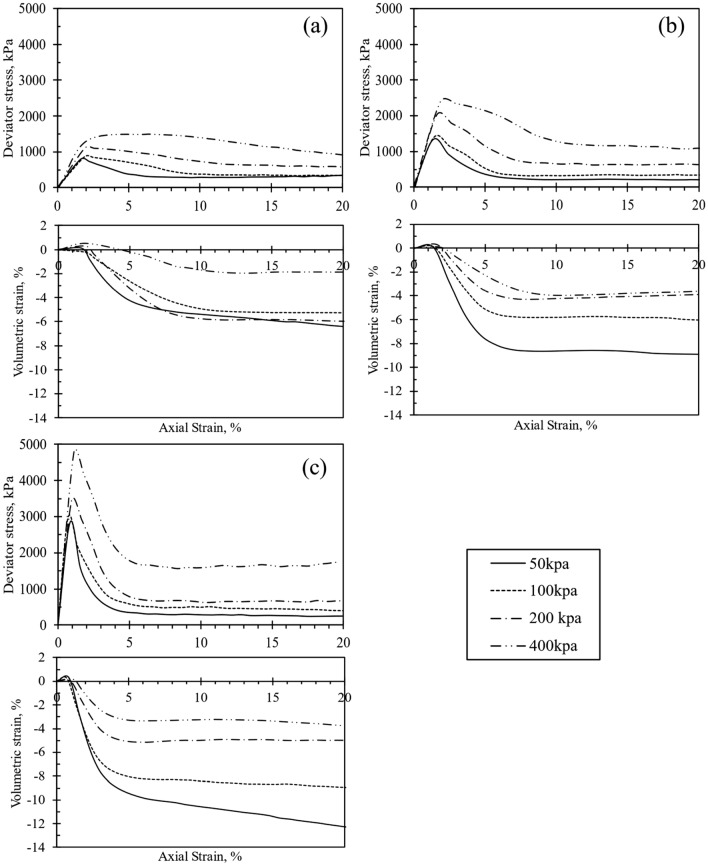
Stress–strain and volumetric-strain curve of CSA cement-treated samples after 14 days of curing, represented for varying cement content: (**a**) 5%, (**b**) 7%, and (**c**) 10%.

**Figure 6:**
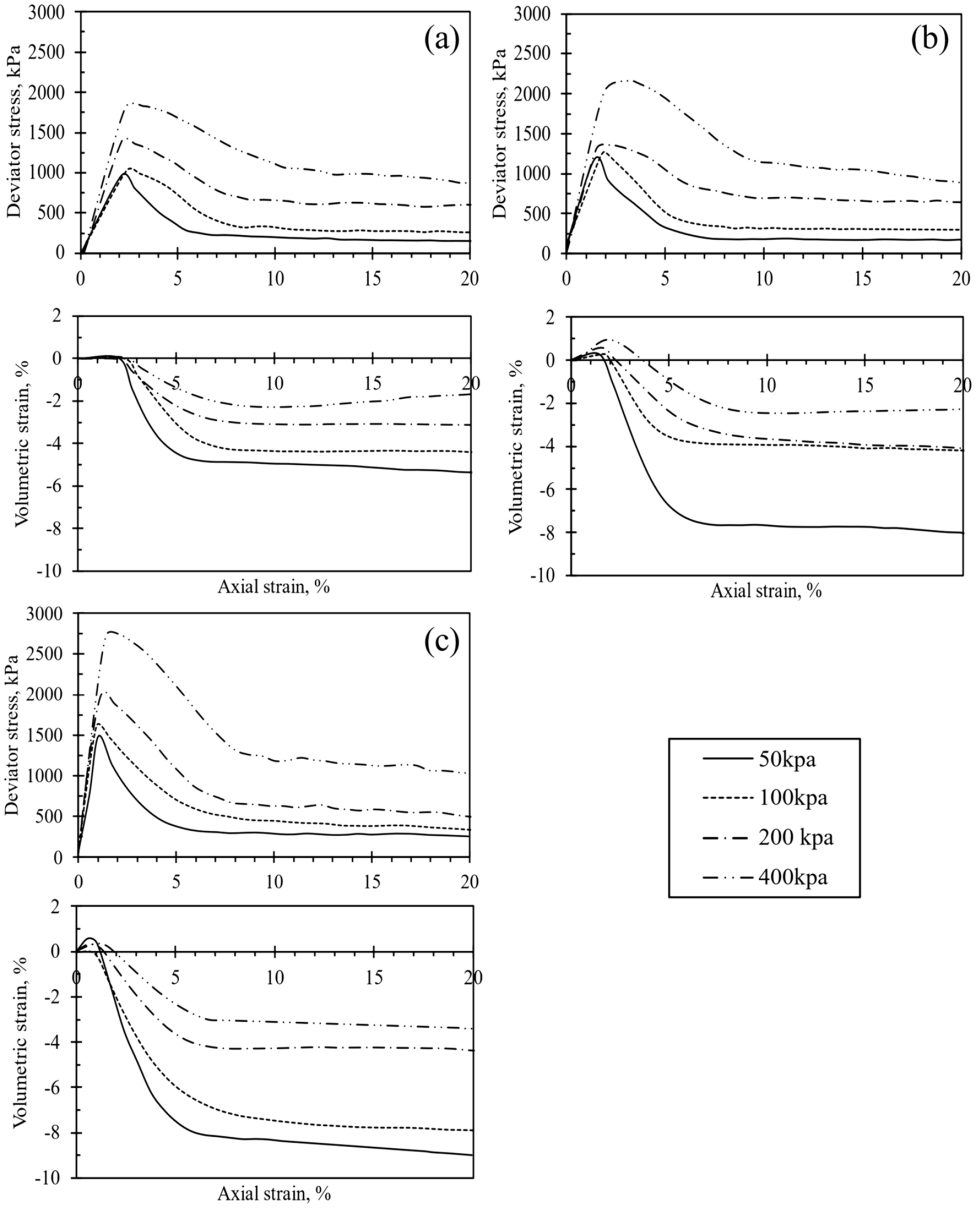
Stress–strain and volumetric-strain curve of 10% OPC cement-treated samples at different curing durations: (**a**) 3 days, (**b**) 7 days, and (**c**) 14 days.

Figures 3, 4, 5 and 6 depict the stress–strain (q-ε_a_) and volumetric strain (ε_v_-ε_a_) behavior of the test samples. Specifically, Fig. 3 through Fig. 5 illustrate the q-ε_a_ and ε_v_-ε_a_ curves of the samples treated with CSA. Notably, an observable trend in these figures indicates an increase in peak deviator stress with increasing cell pressure. Similarly, an increase in cement content corresponded to higher initial stiffness and q_peak_ in the test specimens. However, the rise in CSA cement content was also associated with a reduction in peak axial strain, as depicted in Figs. 3d, 4c, and 5c when 10% of CSA cement content was utilized in sample preparation. Upon reaching q_peak_ during shearing, all test materials treated with CSA cement exhibited strain-softening behavior. Figures 3, 4, 5 further shows that the deviator stresses attained a constant and steady value towards the end of each test, indicating that the tested specimens had reached their ultimate condition. Typically, an increase in the degree of cementation augments q_peak_ reduces the axial strain at failure. Thus, increasing the cement content alters the q-ε_a_ behavior of the cemented specimens from ductile to brittle, as observed by Marri et al.^29^. Furthermore, the q-ε_a_ curves in Figs. 3, 4, 5 indicate that the cement content significantly influences the behavior of CSA-cemented sand. The increase in strength in quartz sand treated with CSA is related to the creation of strong contact bonds between the cement and sand particles, improving its deformation resistance and load-bearing capacity. Furthermore, the test samples treated with 10% OPC exhibited comparable q-ε_a_ behavior to those treated with CSA, as illustrated in Fig. 6.

The ε_v_-ε_a_ curves obtained from the testing of all test samples are presented in Figs. 3, 4, 5 and 6 . Each test sample exhibited an initial phase of volumetric compression followed by dilation. Furthermore, Figs. 3, 4, 5 and 6 indicated that with an increase in cement content, the test materials displayed a more pronounced dilative behavior during shearing. Consequently, under different cell pressures, the compression of cemented samples decreased with higher cement content. The ε_v_-ε_a_ curves also suggested that at the end of each test, the volumetric behavior of the samples approached a constant value. Furthermore, as depicted in Figs. 3, 4, 5 and 6, increasing cementation under low confining pressure diminishes the compression rate of the samples during shearing. Consequently, the influence of both confining pressure and the degree of cementation on the q-ε_a_ behavior of the treated specimens mirrors findings in previous studies^18,22,30^. Additionally, increasing confining pressure in a triaxial test typically enhances q_peak_, ε_a_, and the extension of compression during shearing. Consequently, a surge in cement content raises q_peak_, reduces compression during shearing, and augments dilation.

### Mode of failure

**Figure 7:**
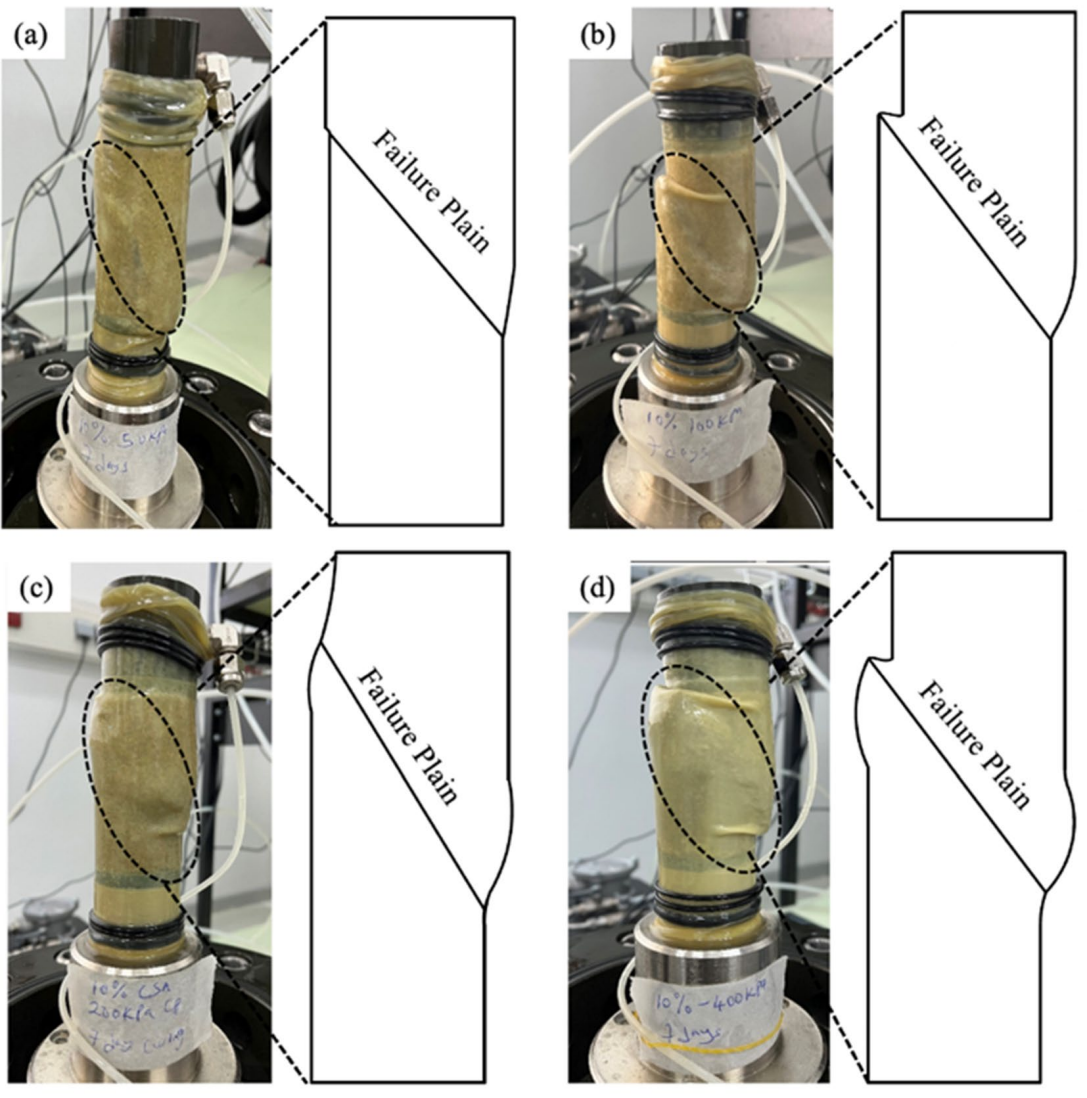
Mode of shear failure and schematics diagram of 10% CSA treated samples sheared at various confining pressures: (**a**) 50 kPa, (**b**) 100 kPa, (**c**) 200 kPa, and (**d**) 400 kPa.

The shear failure mode of a cemented sand is a crucial parameter for analyzing its failure behavior^18,29,30^. The failure mode of the test samples significantly influences the shear strength parameters obtained during the triaxial test. Figure 7 illustrates the failure mode of selected test samples with 10% CSA cement. Generally, the failure mode of sheared cemented samples is characterized by single shear bands without substantial barreling. However, the test sample sheared at a confining pressure greater than 200 kPa exhibited a slight barreling shape with a shear band. This shift is attributed to the increase in confining pressure. The failure mechanism observed in this study aligns with that reported by Amini and Hamidi^22^ for gravelly sand treated with OPC. Figure 7 also shows the schematics diagram of the failure model of the sheared test samples with 10% CSA cement.

### Shear strength parameters

**Figure 8:**
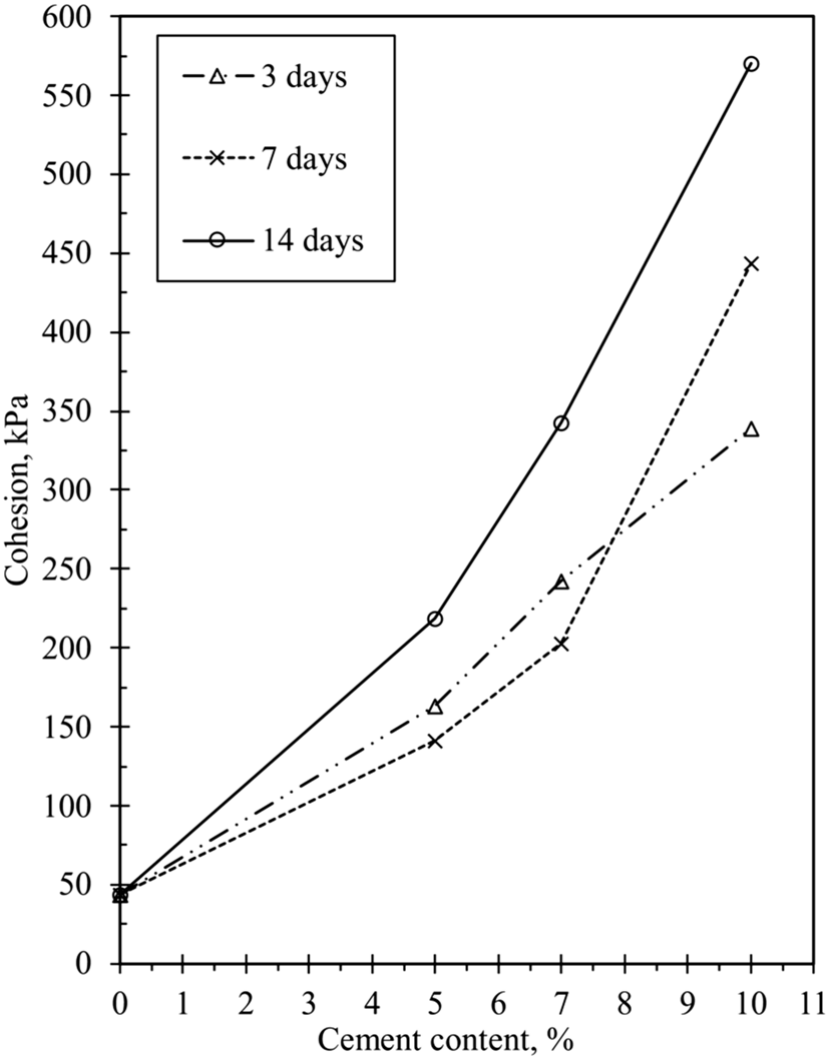
Effect of CSA cement on cohesion intercept.

The shear strength for cemented sand samples depends on cohesion intercept and frictional angle parameters. The correlation between cement content and cohesion intercept is illustrated in Fig. 8, where Mohr–Coulomb diagrams were used to determine frictional angles and cohesion intercepts for samples treated with both OPC and CSA cement. The rise in cement content corresponds to an increase in cohesion among particles in the treated samples, as shown in Fig. 8. Table 2 provides frictional angle and cohesion values derived from the Mohr–Coulomb diagrams. It was observed that samples tested after three days exhibited stronger cohesiveness for confining pressures between 50 to 200 kPa than those tested after seven days. This phenomenon may be attributed to the rapid hydration, faster setting time, and higher early cohesion between the particles in the cemented specimens due to the early strength development characteristics of CSA cement.

**Figure 9:**
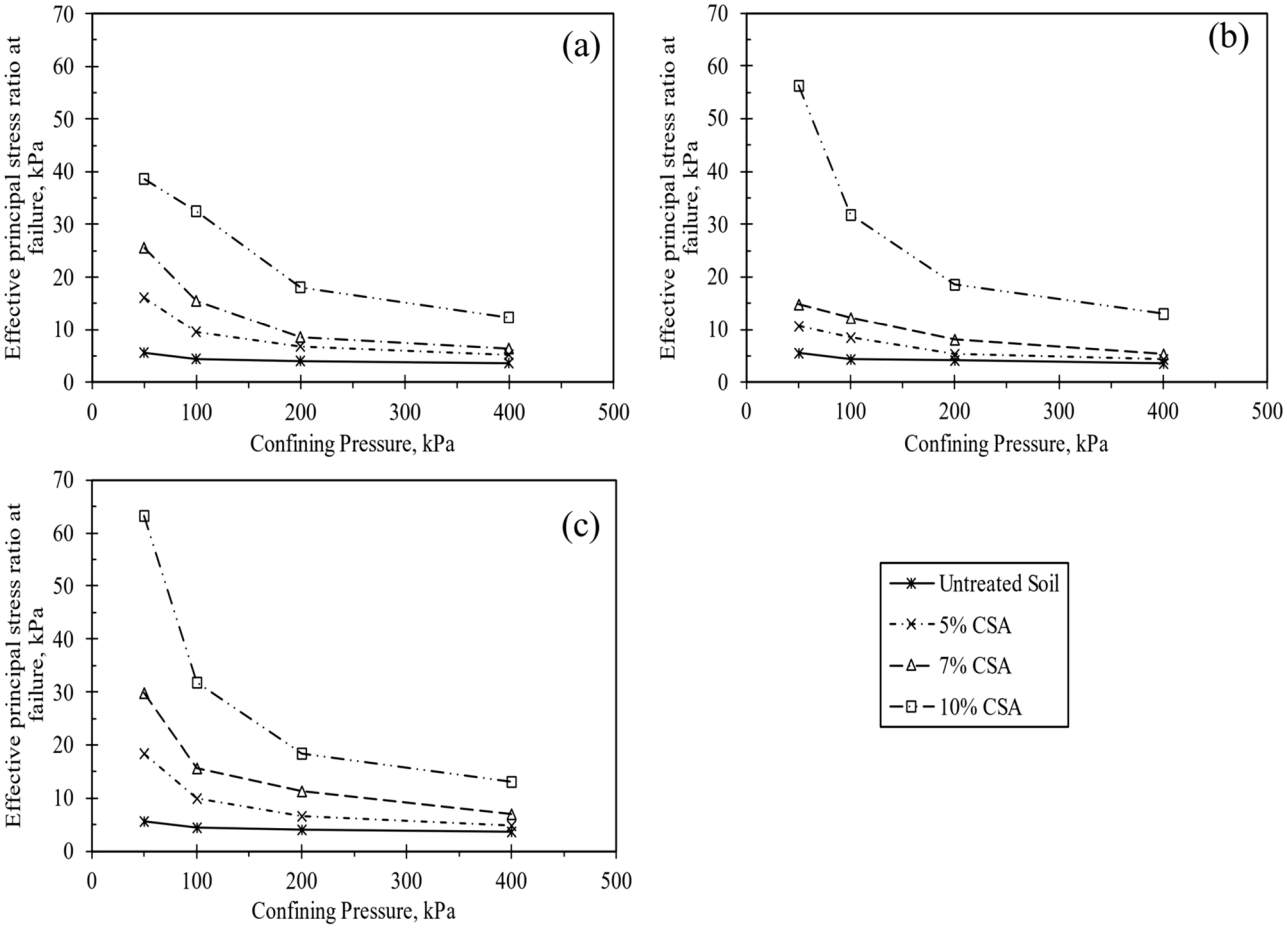
Change in effective principal stress ratio at failure with confining pressure for CSA cement-treated samples at different curing durations: (**a**) 3 days, (**b**) 7 days, and (**c**) 14 days.

Figure 9 further illustrates the variance of the effective principle stress ratio at failure (σ_1_′/σ_3_′)_f_ for treated and untreated test samples. Here, σ_1_′ represents the principle effective stress and σ_3_′ denotes the minor effective stress. According to Fig. 9, the (σ_1_′/σ_3_′)_f_ decreases with increasing degree of cement and confining pressures, indicating substantial effect of both variables on the (σ_1_′/σ_3_′)_f_ of the test specimens.

### Comparative analysis of the mechanical properties of CSA and OPC treated soils

**Figure 10:**
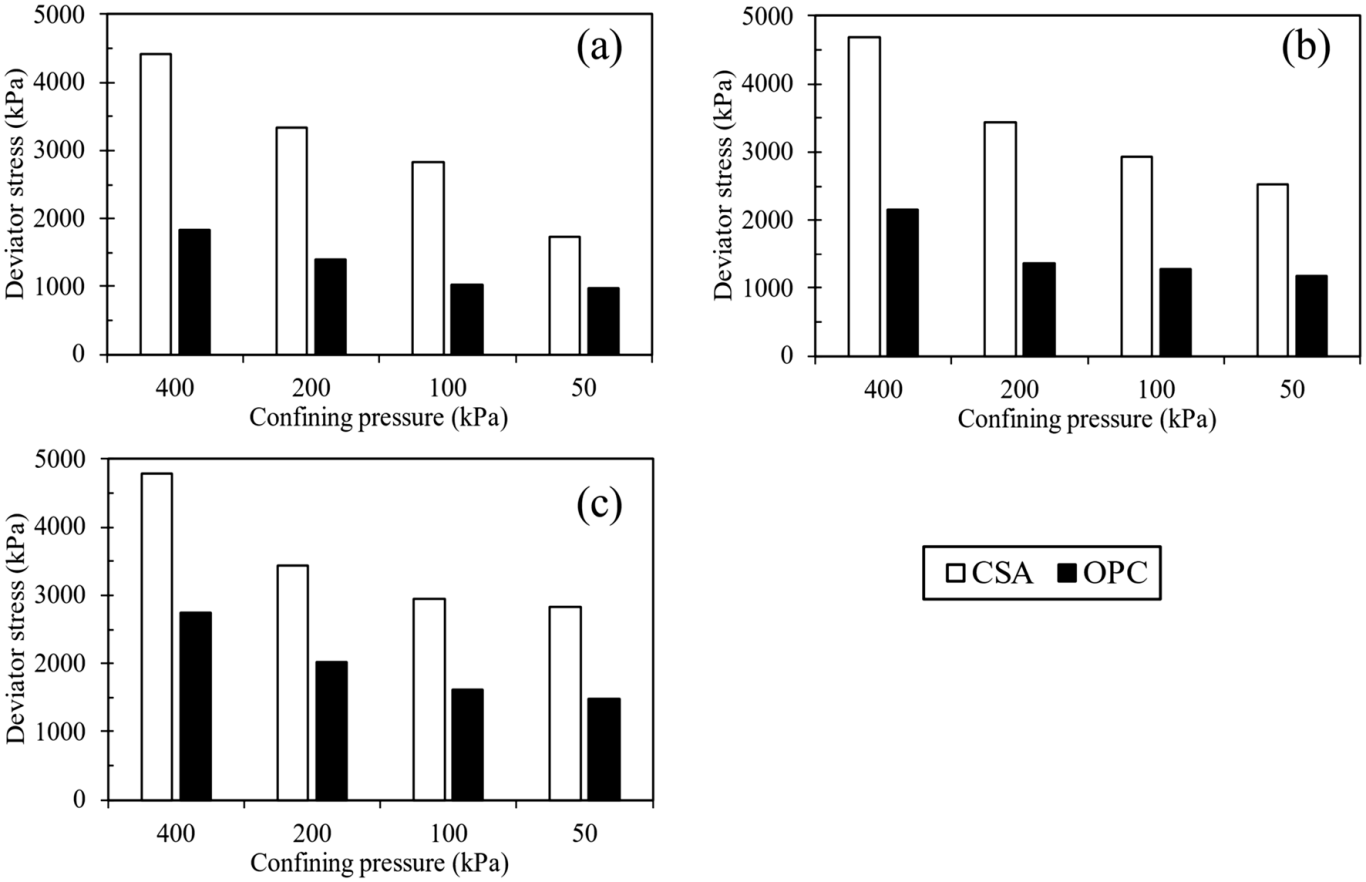
Strength development of the 10% CSA and OPC treated samples at different curing durations: (**a**) 3 days, (**b**) 7 days, and (**c**) 14 days.

Significant trends emerged in the geomechanical characteristics of specimens treated with both CSA and OPC. Figures 3d, 4c, and 5c show the q-ε_a_ and ε_v_-ε_a_ of samples treated with 10% CSA, while Fig. 6 illustrates those treated with OPC and subjected to different curing durations. These figures reveal an augmentation in the q_peak_ and initial stiffness for all cemented samples. Furthermore, a consistent volumetric behavior was observed, with all test specimens exhibiting initial compression followed by dilative during shearing. Regarding strength development, Figs. 3d, 4c, and 5c highlighted that CSA-treated samples exhibited significantly higher peak deviator stress during tests conducted at 3, 7, and 14-day curing periods compared to OPC-treated samples. Additionally, CSA-treated samples demonstrated greater initial stiffness compared to OPC-treated ones. Table 3 summarizes the triaxial test results for both CSA and OPC-treated samples. Figure 10 illustrates the strength development of sand treated with OPC and CSA at 10% cement content and sheared under various confining pressures during testing. These findings underscore the importance of cement type in predicting the mechanical properties of treated sand samples, particularly as CSA cement demonstrates superior strength development and stiffness properties under diverse confining pressures compared to OPC.

**Table 3:** Summary of the test results for 10% CSA and OPC treated samples.

Test identification	Confining pressure, kPa	Curing period, days	Cement content, %	q_peak_, kPa (CSA-treated samples)	q_peak_, kPa (OPC-treated samples)
CD-10/0.05/3	50	3	10	1727	900
CD-10/0.1/3	100	3	10	3032	1002
CD-10/0.2/3	200	3	10	3329	1394
CD-10/0.4/3	400	3	10	4484	1828
CD-10/0.05/7	50	7	10	2525	1182
CD-10/0.1/7	100	7	10	3106	1276
CD-10/0.2/7	200	7	10	3429	1362
CD-10/0.4/7	400	7	10	4735	2155
CD-10/0.05/14	50	14	10	2837	1481
CD-10/0.1/14	100	14	10	2955	1607
CD-10/0.2/14	200	14	10	3444	2018
CD-10/0.4/14	400	12	10	4792	2757

**Figure 11:**
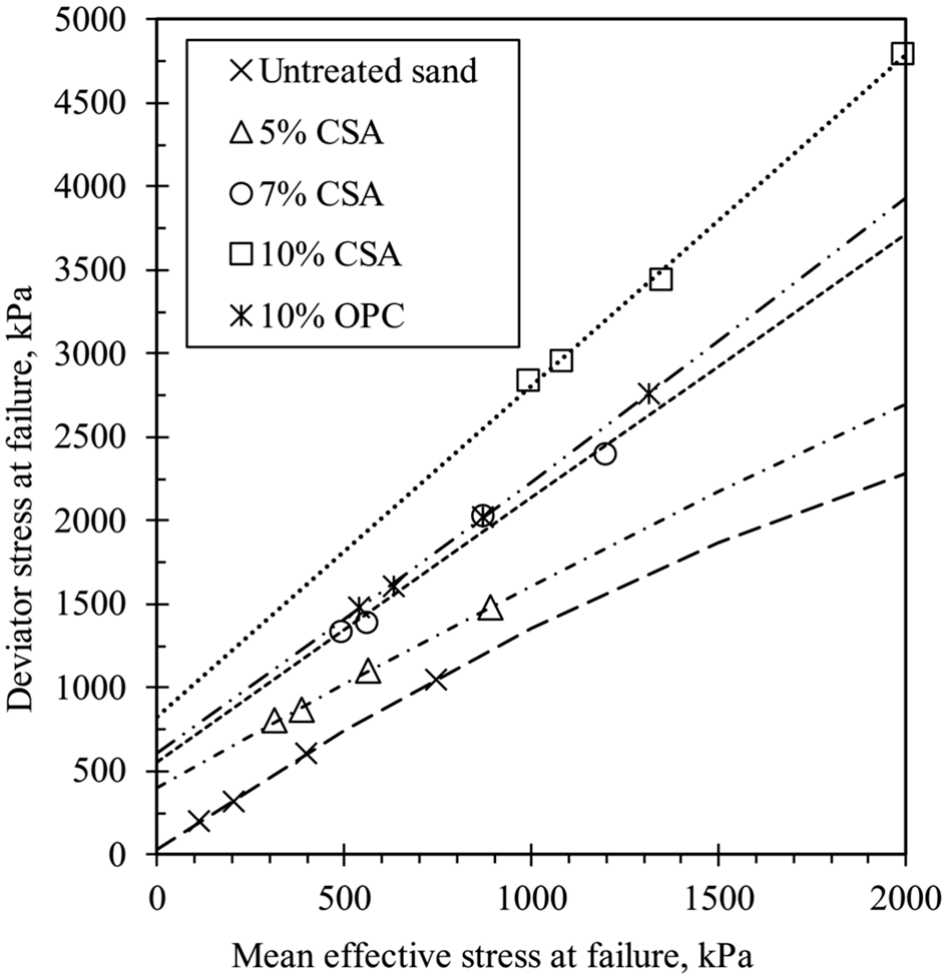
Failure envelopes of cemented and uncemented sand for 14 days curing.

**Figure 12:**
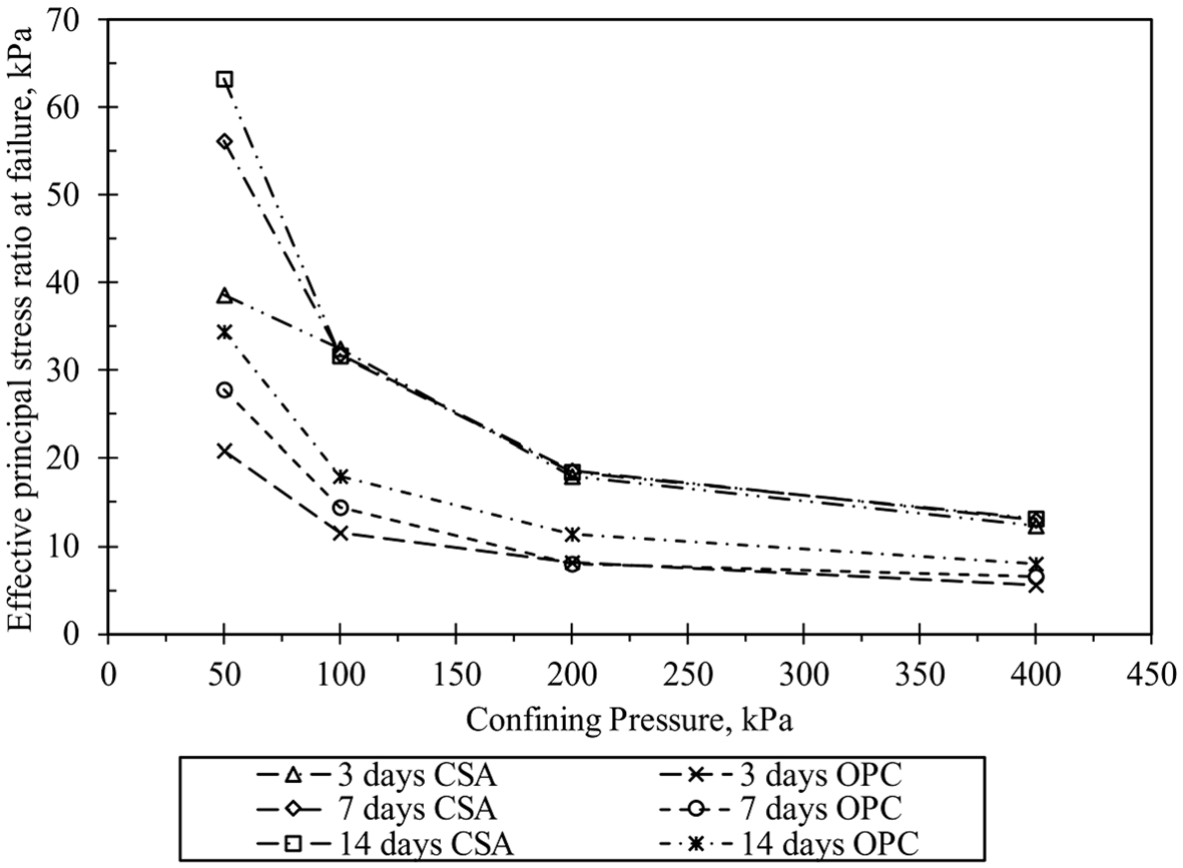
Change in effective principal stress ratio at failure with confining pressure for 10% CSA amd OPC cement-treated samples at different curing durations.

Figure 11 presents the stress path (q-p′) for the specimens treated with cement after 14 days of curing. The figure depicts curved failure envelopes for the tested specimens, and a polynomial function was used for the line of best fit. The curved failure envelopes align with previous findings from researchers conducting triaxial testing on cemented sand at high confining pressure^26,29,31^. Additionally, the curvature of the failure envelopes increases with the degree of cementation. Figure 11 indicates that the failure envelopes shift toward higher stress levels as the amount of CSA cement increases, suggesting that higher cement content enhances cohesion among the sand particles. Moreover, the figure illustrates that higher cement content transforms the failure envelope from non-linear to linear. This change is attributed to increased cohesion resulting from cement addition. Furthermore, Fig. 11 demonstrates that the slopes of failure envelopes decrease with rising confining pressure, highlighting the influence of confining pressure on the failure envelope of cement-treated sand. Figure 12 shows the variance of the effective principle stress ratio at failure (σ_1_′/σ_3_′)_f_ for 10% CSA and OPC-treated samples. It can be observed from the figure that CSA-treated samples had higher (σ_1_′/σ_3_′)_f_ than OPC samples. This is due to the strong contact bonds and higher frictional angle exhibited by the samples treated with CSA cement.

**Figure 13:**
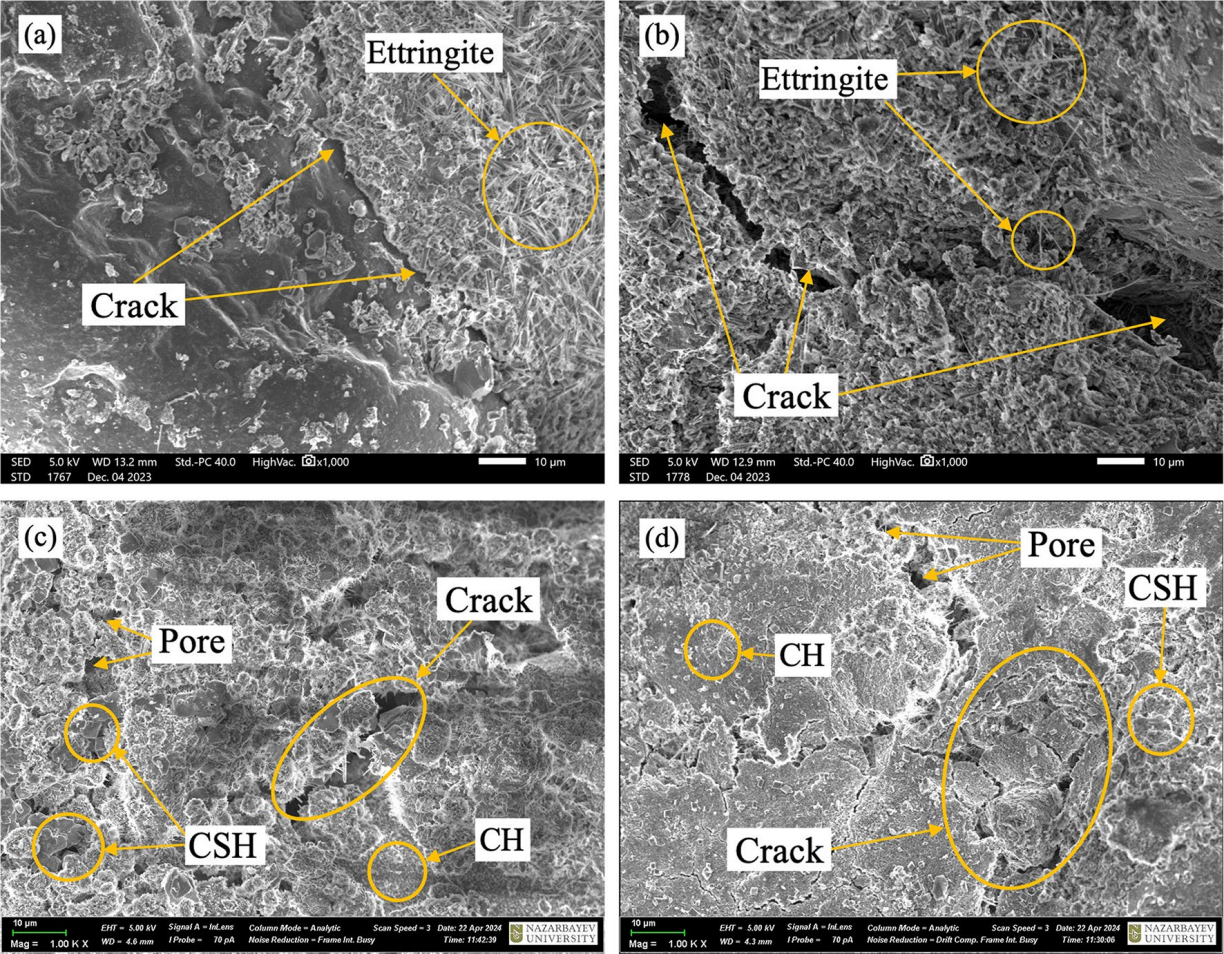
SEM images of soil specimens treated with 10% CSA cement and OPC for 3 days curing period under different confining pressures: (**a**) 50 kPa and (**b**) 400 kPa for CSA cement; (**c**) 50 kPa and (**d**) 400 kPa for OPC.

After the triaxial test, the sheared cemented samples underwent SEM examination to evaluate sample deformation, cement bond failure, and particle breakage. SEM images were collected for four separate test samples (two for CSA and OPC samples) sheared at 50 and 400 kPa, respectively. Figure 13a, b illustrate the impact of confining pressure on particle breakage and bond failure in CSA-treated materials, highlighting ettringite formation due to the hydration of calcium sulfoaluminate minerals. These findings aligns with a previous study on cemented sand by^18,29^, showing that bond breaking and particle crushing in CSA-treated materials increase with confining pressure. In contrast, samples treated with OPC displayed a much lower response to confining pressure, attributed to the presence of unhydrated particles and a slower hydration rate compared to CSA cement. Figure 13c, d further demonstrate that as confining pressure increases from 50 to 400 kPa, both particle breakage and cement bond intensify. Additionally, these figures reveal that calcium hydroxide (CH) and calcium silicate hydrate (CSH), with its characteristic sheet-like structure, are the predominant hydration products of OPC-treated samples.

### Brittleness index

According to Consoli et al.^39^, the brittleness index (IB) can be employed to assess soil brittleness. The equation below can be used to calculate IB.

4 $${\text{IB}} = \frac{{{\text{q}}_{{{\text{max}}}} }}{{{\text{q}}_{{{\text{res}}}} }} - 1$$

**Figure 14:**
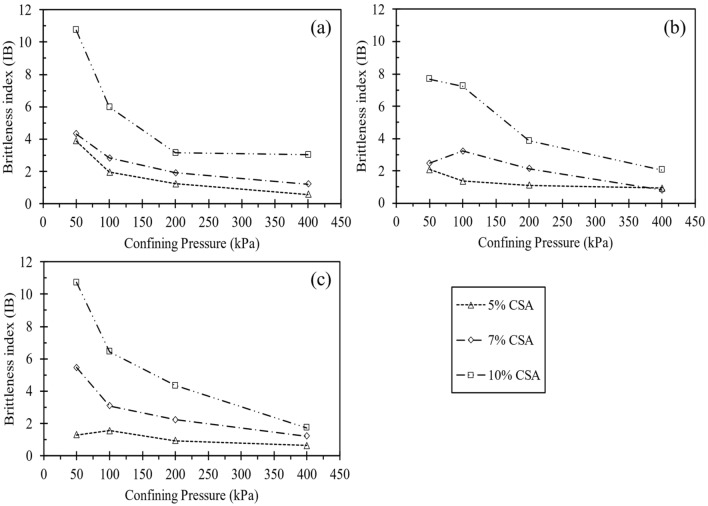
Variation in the brittleness index under different confining pressures at different curing durations: (**a**) 3 days, (**b**) 7 days, and (**c**) 14 days.

**Figure 15:**
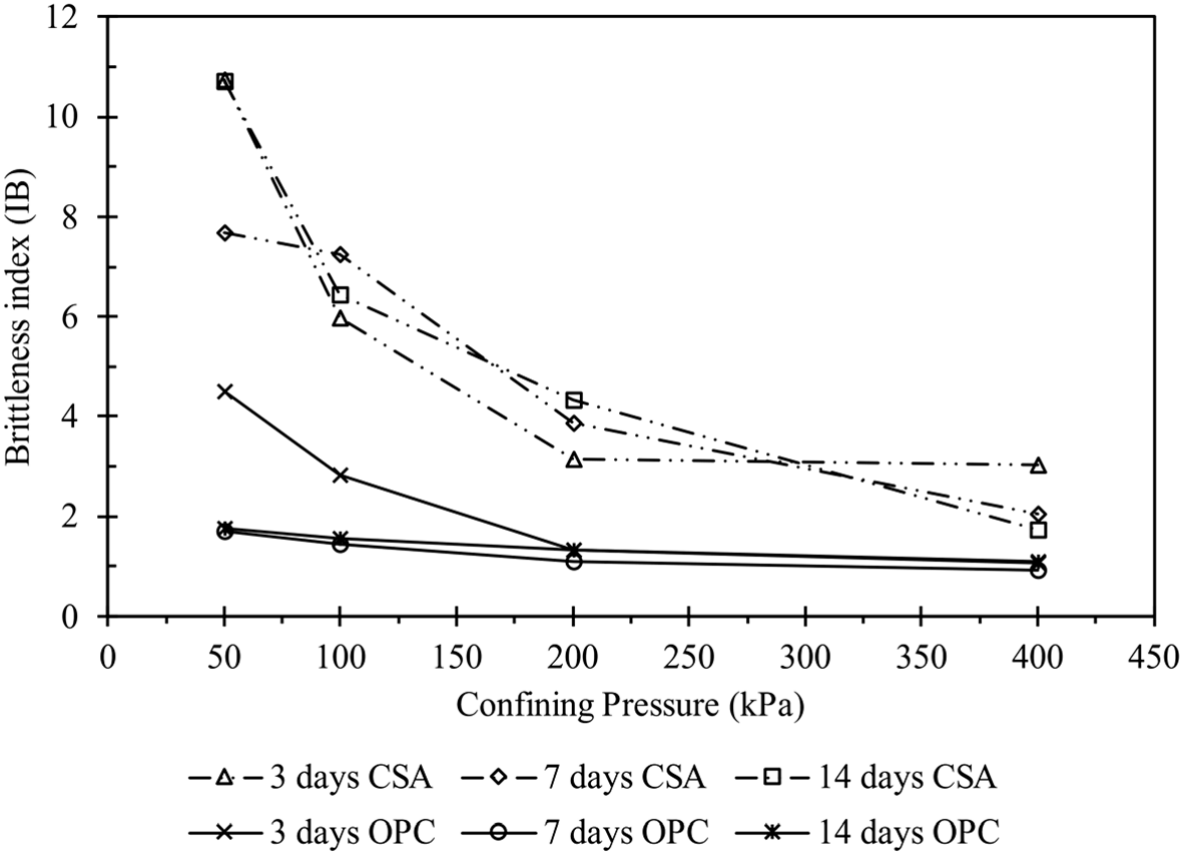
Variation in the brittleness index under different confining pressures at different curing durations for 10% CSA and OPC cement-treated samples.

The brittleness index, calculated from the triaxial testing results, is shown in Fig. 14, where q_res_ and q_max_ represent residual and peak deviator stress, respectively. As observed in Fig. 14, the brittleness index increases with relative density and decreases with increased confining pressure. Consequently, the cemented samples exhibit increased brittleness as the confining pressure rises. Figure 15 illustrates the brittleness index for 10% of both CSA- and OPC-treated samples. It can be observed from the figure that CSA-treated samples had a higher brittleness index for all cement content. This disparity can be attributed to the higher frictional angle and cohesion evident in the CSA-treated samples.

### Stiffness and energy absorption

**Figure 16:**
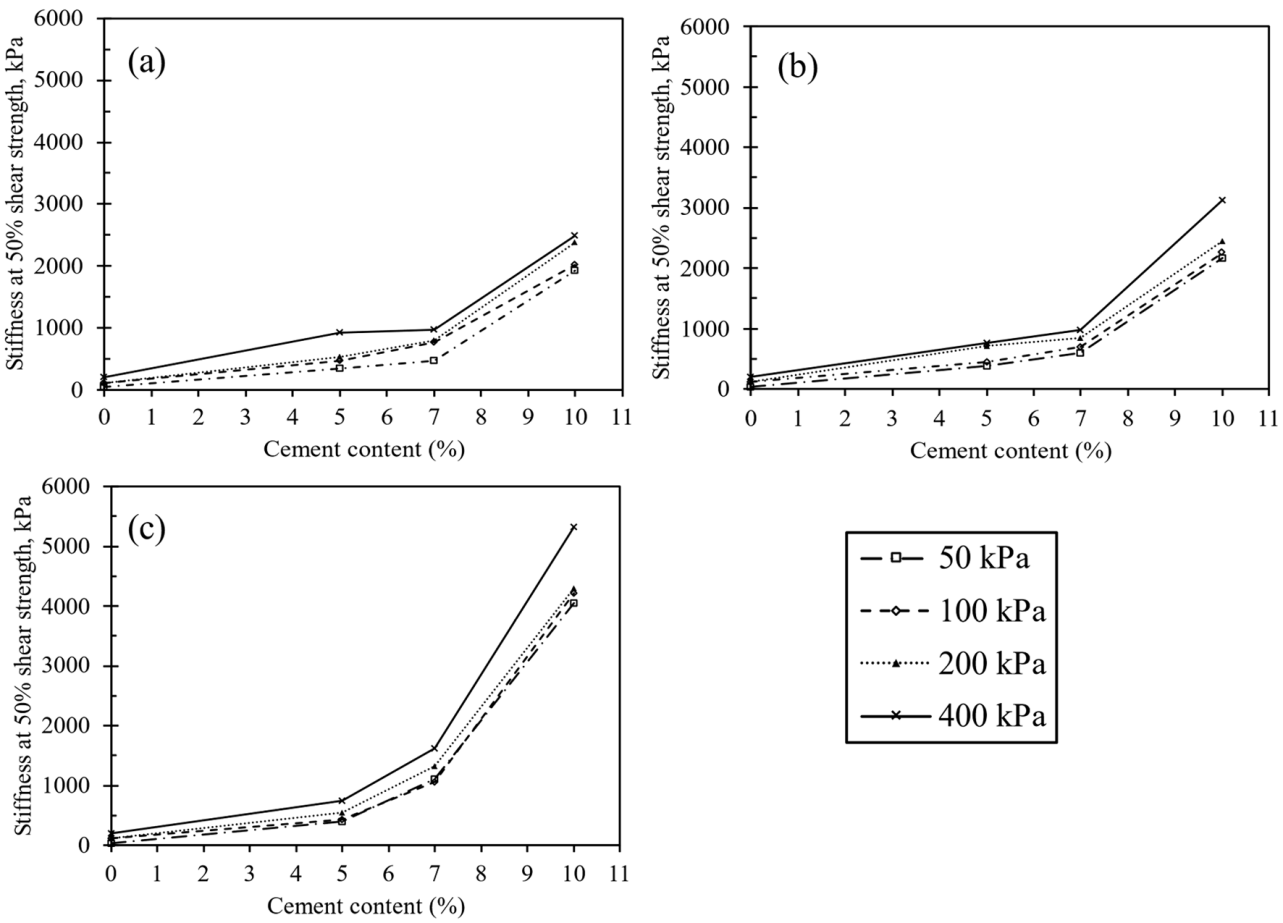
Changes in stiffness under various confining pressures for CSA cement at different curing durations: (**a**) 3 days, (**b**) 7 days, and (**c**) 14 days.

Figure 16 illustrates the determination of the secant modulus for half the shear strength at various confining pressures to ascertain the stiffness of the treated specimens. The correlation between confining pressure, stiffness of the treated sand, and cement content is also presented in Fig. 16. The figure also highlighted the importance of cementation and confining pressure for soil improvement. As depicted in the figure, an increase in confining pressure and cement content corresponds to an increase in the stiffness of the cemented specimen.

**Figure 17:**
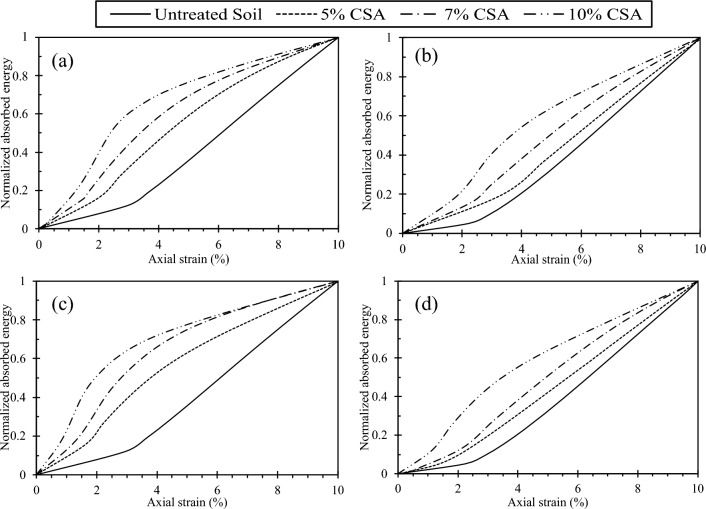
Correlation between normalized absorbed energy and cement content across different confining pressures (CP) and curing durations: (**a**) CP = 50 kPa, 7 days curing, (**b**) CP = 400 kPa, 7 days curing, (**c**) CP = 50 kPa, 14 days curing, and (**d**) CP = 400 kPa, 14 days curing.

**Figure 18:**
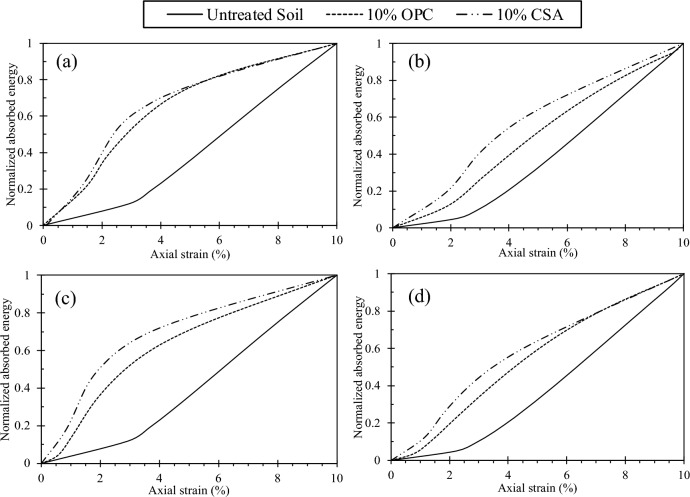
Correlation between normalized absorbed energy and cement content across different confining pressures (CP) and curing durations: (**a**) CP = 50 kPa, 7 days curing, (**b**) CP = 400 kPa, 7 days curing, (**c**) CP = 50 kPa, 14 days curing, and (**d**) CP = 400 kPa, 14 days curing.

Similarly, the energy required to induce deformation in the test samples, commonly referred to as energy absorption, was determined. It was assessed by calculating the area under the q-ε_a_ curve. The energy absorption for all tests conducted in this study was evaluated at an axial strain of 10%. Figure 17 displays the relationship between normalized absorbed energy for CSA-treated samples tested at 7 and 14 days of curing under 50 kPa and 400 kPa confining pressure. Additionally, Fig. 17 reveals that test materials with a higher relative density require more energy to deform. These findings indicate that samples with higher relative density before deformation absorb more energy as the confining pressure increases. Figure 18 depicts the normalized absorbed energy for samples treated with 10% CSA and OPC, tested at 7 and 14 days of curing under 50 kPa and 400 kPa confining pressures. The figure highlights that CSA-treated samples exhibit a higher energy requirement for deformation than OPC-treated samples. This distinction can also be ascribed to the greater frictional angle and cohesion exhibited by the CSA-treated samples.

## Conclusion

In summary, this research underscores the effectiveness of calcium sulfoaluminate (CSA) cement in enhancing the geomechanical characteristics of granular soil, particularly under low confining pressures. Moreover, the study highlights the pronounced environmental benefits and superior performance of CSA cement in comparison to conventional ordinary Portland cement (OPC), particularly concerning early strength development, shear strength, and deformation parameters. The key conclusions drawn from the test results are as follows:

1. Shear strength enhancement: Increased CSA cement not only enhanced shear strength and stiffness in treated sand but also elevates peak deviator stress while reducing compression during shearing. Higher confining pressure further intensifies peak deviator stress and compression, rendering cemented samples brittle at elevated confining pressure and cement content.
2. Influence of cement type and content: Cement type and content exert a substantial impact on the cohesive and frictional properties of cemented sand. The failure envelopes underscore the critical role of these variables in soil improvement.
3. Failure mode characteristics: Analysis of stress–strain curve and failure mode images reveals that sheared cemented samples predominantly exhibit single shear bands with limited barreling. However, as incremental increase in confining pressure leads to a gradual transition in the failure mode from brittle to slightly ductile.
4. SEM analysis insights: SEM analysis provides valuable insights into the microstructure of cemented sand under varying confining pressures, focusing on bond degradation and particle compression processes. The degree of particle breakage during shearing is obserbed to increase with confining pressure.
5. Sustainable geotechnical engineering: This research makes a significant contribution to sustainable geotechnical engineering by showcasing the environmental advantages of CSA cement and its feasibility as a material for enhancing soil properties, especially under low confining pressures conditions.

## Data Availability

The datasets used and/or analysed during the current study available from the corresponding author on reasonable request.
